# Injection Molding Condition Effects on the Mechanical Properties of Coconut-Wood-Powder-Based Polymer Composite

**DOI:** 10.3390/polym16091225

**Published:** 2024-04-27

**Authors:** Quach Van Thiem, Van-Thuc Nguyen, Dang Thu Thi Phan, Pham Son Minh

**Affiliations:** Faculty of Mechanical Engineering, Ho Chi Minh City University of Technology and Education, Ho Chi Minh City 71307, Vietnam; thiemqv@hcmute.edu.vn (Q.V.T.); nvthuc@hcmute.edu.vn (V.-T.N.); thuptd@hcmute.edu.vn (D.T.T.P.)

**Keywords:** wood powder, tensile strength, flexural strength, optimization, injection molding

## Abstract

This study investigates the mechanical properties of coconut sawdust powder combined with polypropylene (PP). The effect of compatibility content, wood powder (WP) content, and injection molding parameters on the properties of coconut wood powder composite (WPC) is evaluated. The results could be used to figure out the optimal mechanical properties such as tensile strength, elongation, elastic modulus, and flexural strength by selecting suitable parameters and composition. The bonding between the WP particles and the PP matrix is good, and the WP is uniformly distributed across the composite matrix, as indicated in the scanning electron microscopy (SEM) results. Interestingly, with the presence of the compatibilizer oleamide, increasing the WP content from 20 wt.% to 40 wt.% did not result in WP accumulation in the composite matrix. Notably, at 20 wt.% WP, the elongation is the highest (at 7.40 wt.%), while at 30 wt.% WP, the elastic modulus reaches the highest value. The maximum ultimate tensile strength (UTS) value is obtained at 35 wt.% WP. Higher WP mostly results in greater flexural strength and shore D hardness. At 40 wt.% WP, the WPC achieves its peak shore D hardness of 77.6. The Taguchi results suggest that WP content is the most critical factor in the UTS value of coconut WPCs. The filling pressure ranks second, followed by the packing pressure. Finally, unlike the other characteristics, the melt temperature has a minimal impact on the UTS value.

## 1. Introduction

Recently, exploring ways to reuse waste products has become an attractive method of reducing pollution, saving energy, and saving material resources [1,2]. For instance, wood-powder-based composites have become a trend in green policies to reduce the use of plastics [3,4,5,6,7]. The wood powder (WP) is the bypass product, collected from the process of wood production. Wood products can be burned to create heat sources. WP can also be combined with some polymers to create a wood-based composite [8,9,10,11]. WP can also be mixed with ceramics to generate ceramic composites [12,13,14,15,16].

Dikobe et al. [17] investigated the morphology, mechanical properties, and thermal properties of the WP composite with polypropylene (PP)/linear low-density polyethylene (LLDPE) and maleic anhydride-grafted polypropylene (MAPP)/LLDPE blend systems. The composite used 10, 20, and 30 wt.% WP. The MAPP, LLDPE, and WP composites have stronger interfacial bonding and better thermal stability than the PP, LLDPE, and WP composites. Nygård et al. [18] improved the feeding ability of the wood fiber during the extrusion process. They showed that pelletizing greatly reduced the length of wood fibers while allowing for a regulated feeding of the fibers into the extruder. Buddi et al. [19] reported the mechanical properties of a 5 wt.% WP and HDPE composite enhanced with nanoSiO_2_. With 5 wt.% SiO_2_, the composite reached the highest tensile and flexural strengths. The higher SiO_2_ content led to the segregation of nanoparticles and a reduction in the ductility of the composite. The composite obtained the highest impact strength at 3 wt.% SiO_2_. He et al. [20] mixed WP with polyvinyl alcohol (PVA) by applying solid-state shear milling and thermal processing, allowing for the high productivity of the composite. The composites achieved a good tensile strength of 22.5 MPa and elongation of 120.5 wt.%, which is greatly higher than the traditional mixing method.

Furthermore, wood-powder-based composite (WPC) could be processed by various technologies such as extrusion, injection, or compression molding [21]. WPC products can appear in automobiles, construction, and furniture due to the advantages of weather duration and biological impact. Notably, da Silva et al. [22] reported that polylactic acid (PLA)/poly ε-caprolactone (PCL) composites combined with WP. The initial mixture is mixed by a screw extruder, graded, and finally injected by an injection molding machine. The tensile strength and elastic modulus of the composites are reduced; however, the impact strength of the composite is higher than that of pure PLA. The composite material is good enough to generate disposable cups. Interestingly, the WPC could be used as a 3D printing material [23]. Huang et al. [24] mixed WP, acrylonitrile–butadiene–styrene (ABS) polymer, and maleic anhydride to create 3D printing materials. They found that the best percentage of Red Gum WP in the composites is 29 wt.%.

For a denser and stronger 3D-printed WPC product, it is suggested to use wood particles with more rounded shapes and smoother surfaces. Kajaks et al. [25] mixed coupling-agent-maleated polypropylene (MAPP) with WP. The use of a coupling agent significantly increases the tensile strength, flexural strength, and impact strength by up to 1.5–2.2 times. The mechanical properties of high-density polyethylene (HDPE) mixed with WP are also surveyed [26]. The WP percentage could reach 50 wt.%, while the mechanical properties are still good enough. The best water resistance is obtained at 5 wt.% MAPP. Scanning electron microscopy (SEM) results showed that MAPP increases the interfacial bonding between the WP and HDPE matrix. Salemane and Luyt [27] combined PP and WP with some compatibilizers to create WPC. The findings suggest that WP settles in the amorphous component of the matrix, forming new crystalline phases or zones. SEM micrographs reveal a relatively equal distribution of WP in the PP matrix, which contributes to reported improvements in material characteristics.

Most of the above studies did not mention the impact of the injection molding parameters on the characteristics of the WPC. Moreover, the coconut sawdust by-products are also rarely discussed when exploring the WPC. Coconut sawdust is a waste product which is popular in South and South East Asia. Finding the optimal ways to reuse it could minimize pollution, save energy, and save materials and resources [1,2,3,4,5]. This report concentrates on studying the mechanical properties of coconut sawdust powder mixed with PP. The WP is dried and then mixed with PP and additives, following an extrusion process to generate granular. The composite is then injected by an injection molding machine to create the test samples. The impact of compatibility content, WP content, and injection molding parameters on the characteristics of the coconut WPC are examined. The WPC samples are tested via tensile, flexural, and hardness tests. The samples are also tested with a water absorption test, a scanning electron microscope (SEM) test, a shore D hardness test, a water absorption test, and a differential scanning calorimetry (DSC) test. The optimal tensile strength, elongation, elastic modulus, tensile strength, and hardness are indicated. The results might be used to determine the best mechanical qualities, such as tensile strength, elongation, elastic modulus, and flexural strength, by choosing the appropriate parameters and composition.

## 2. Experimental Methods

### Materials and Method

**Figure 1:**
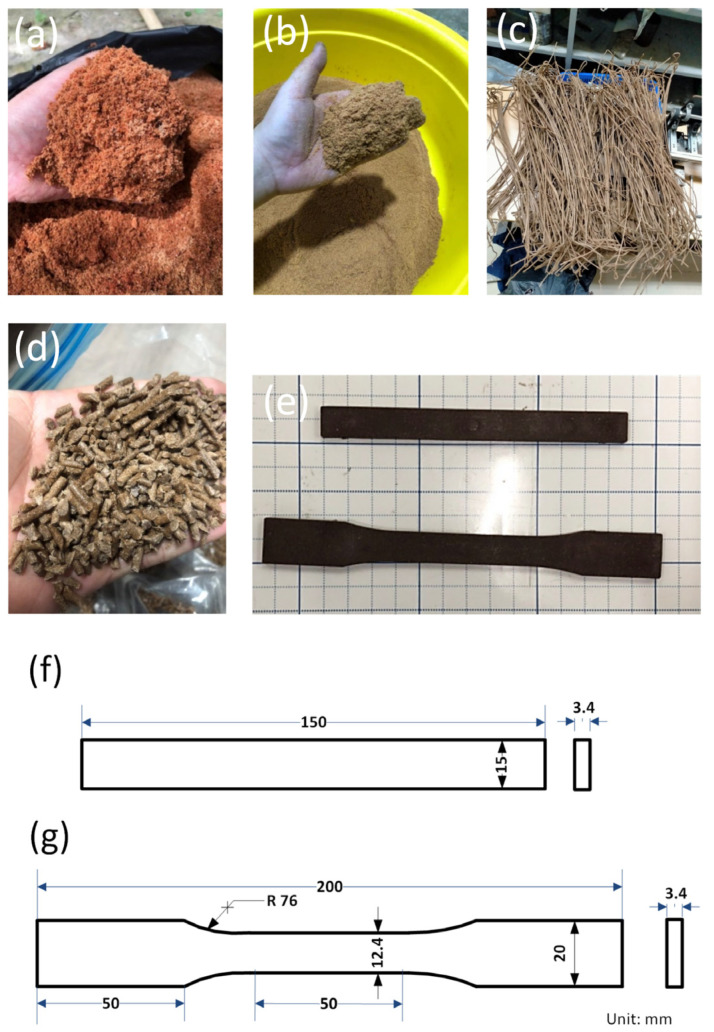
Sample preparation process: (**a**) coconut WP before filtering; (**b**) coconut WP after filtering; (**c**) coconut WP and PP after extrusion; (**d**) granulation; (**e**) tensile and flexural injection samples; (**f**) flexural sample dimension; (**g**) tensile sample dimension.

Figure 1a,b show the coconut sawdust powder before and after filtering by a fine mesh with a 60-mesh US standard. Then, it is dried at 90 °C for 8 h to remove humidity [28,29]. The WP is then mixed with PP polymer, 5 wt.% compatibilizer, and 2 wt.% lubrication additive for 30 min in a mixer. Oleamide O2136 lubrication additive is supplied by MERCK Company, Darmstadt, Germany. Compatibilizer Scona TPPP 8112 GA is from BYK Kometra GmbH, Schkopau, Germany. A PP polymer named Advanced-PP 1100 N is supplied by Advanced Petrochemical Company in Al Jubail 35725, Saudi Arabia, and its characteristics are shown in Table 1. The coconut WP in the WPC varies from 20 wt.% to 40 wt.%. After that, the mixture is extruded at 160 °C and a screw rotation speed of 150 rpm and granulated to create composite granulation with the size of 3.2 mm × 5 mm, as shown in Figure 1c,d. The composition granulation is dried for 8 h at 90 °C to remove humidity before being injected into the injection molding machine Haitian-MA 1200III at different injection parameters. Each WPC composition has eight samples: four samples for the tensile test and four samples for the flexural test. In Section 3.1 and Section 3.2, the WPC is injected at a filling pressure of 33 bar, a packing pressure of 40 bar, and a melt temperature of 220 °C.

The WPC samples are injected following ASTM D638 for the tensile test and ASTM D790 for the flexural test [38,39]. After being injected, the samples are subjected to tensile and flexural tests by a tensile test machine AG-X Plus 20 kN (Shimadzu, Kyoto, Japan) at a 5 mm/min speed. The fracture surfaces are examined via an SEM microscope TM4000 (Hitachi, Tokyo, Japan) with a voltage of 5 kV, magnification of ×100, working distance of 6.2–7.7 mm, and an emission current of 65,200–67,900 nA. The samples are also measured with DSC via DSC 214 Polyma (NETZSCH, Selb, Germany) to indicate the crystallinity percentage at a range of 40–250 °C, a speed of 10 K/min, and atmosphere of N2, 40.0–60 mL/min. In addition, the samples are also measured with shore D hardness tester LX-D (ETOPOO, Zhejiang, China). Finally, the water absorption is tested via standard ASTM D 570-88, using digital scale FA2204 (JOANLAB, Huzhou, China).

## 3. Results and Discussion

### 3.1. Tensile Test Results

**Figure 2:**
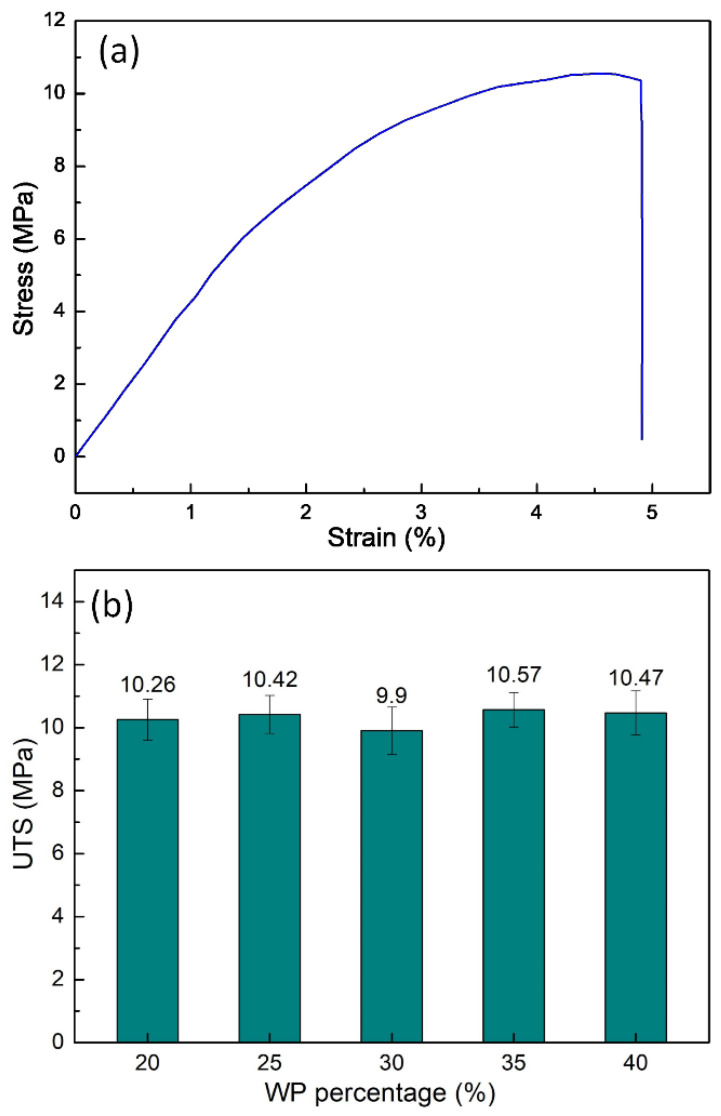
Stress–strain diagram and UTS comparison of WPC at different WP percentages: (**a**) stress–strain diagram of sample with 20 wt.% WP; (**b**) UTS comparison diagram.

Figure 2 shows the stress–strain diagram of the WPC at different WP percentages. The UTS values of the samples are 10.26 MPa, 10.42 MPa, 9.90 MPa, 10.57 MPa, and 10.47 MPa, corresponding to 20 wt.%, 25 wt.%, 30 wt.%, 35 wt.%, and 40 wt.% WP. The UTS value of the WPC varies from 9.90 MPa to 10.57 MPa. This result shows that changing the WP content does not strongly impact the UTS value of the WPC samples. Moreover, the highest UTS value is 10.57 MPa, which is achieved with 35 wt.% WP. With the WP from sanding dust birch wood veneer plywood in the PP matrix, Kajaks et al. [25] indicated that increasing from 20 wt.% to 40 wt.% WP does not strongly change the UTS value, which is consistent with this report.

**Figure 3:**
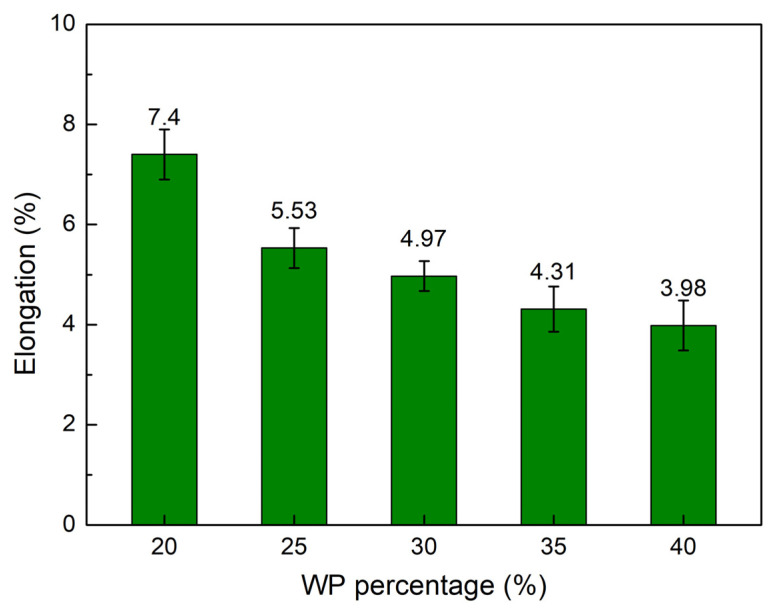
Elongation values comparison of WPC at different WP percentages.

The elongation at break comparison diagram of the WPC at different WP percentages is presented in Figure 3. The elongation values of the WPC samples are 7.40 wt.%, 5.53 wt.%, 4.97 wt.%, 4.31 wt.%, and 3.98 wt.%, corresponding to 20 wt.%, 25 wt.%, 30 wt.%, 35 wt.%, and 40 wt.% WP. The results indicate that increasing the WP content mostly leads to decreasing the elongation at break value. By limiting the mobility of the polymer chains, the presence of WP in the matrix reduces the sample’s ability to deform [27]. WPC with 20 wt.% WP has the highest elongation value of 7.40 wt.%, while WPC with 35 wt.% WP obtains the lowest elongation value of 4.31 wt.%. Furthermore, the elongation at the break value of the WPC samples is smaller than the original PP polymer due to the low elongation of the WP. Kajaks et al. [25] also indicated the reduction of the elongation at break value when increasing the WP content from 20 wt.% to 40 wt.%. The reduction value is minor due to the existence of the compatibilizer in the WPC.

**Figure 4:**
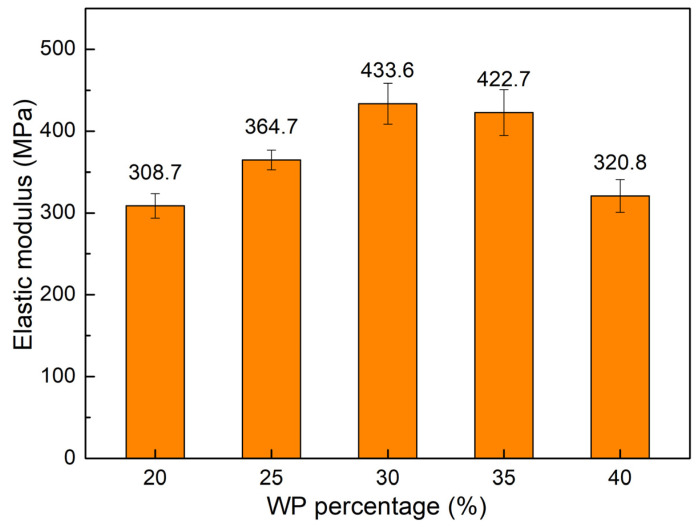
Elastic modulus values comparison of WPC at different WP percentages.

Figure 4 shows the elastic modulus value comparison of WPC at different WP percentages. The elastic modulus values are 308.7 GPa, 364.7 MPa, 433.6 MPa, 422.7 MPa, and 320.8 MPa, corresponding to 20 wt.%, 25 wt.%, 30 wt.%, 35 wt.%, and 40 wt.% WP. Different from the elongation diagram, which goes down and then rises when increasing the WP content, the elastic modulus diagram goes up and then goes down. The reason is the reverse characteristic of the elongation and elastic modulus values. From 20 wt.% to 30 wt.% WP, with compatibilizer, there is a strong interaction between the PP and WP particles. This will reduce chain mobility and increase the elastic modulus with increasing WP content. On the other hand, the increase in WP could lead to the accumulation of WP, leading to a reduction in the elastic modulus due to the coarsening of WP. With 30 wt.% WP, the WPC reaches the highest value of elastic modulus at 433.6 MPa. On the contrary, with 20 wt.% WP, the WPC has the lowest elastic modulus at 308.7 MPa. Salemane et al. [27] also figured out that increasing the WP content will first lead to an increase in the elastic modulus, and then, further increases in the WP will result in a reduction in the elastic modulus of the WPC.

**Figure 5:**
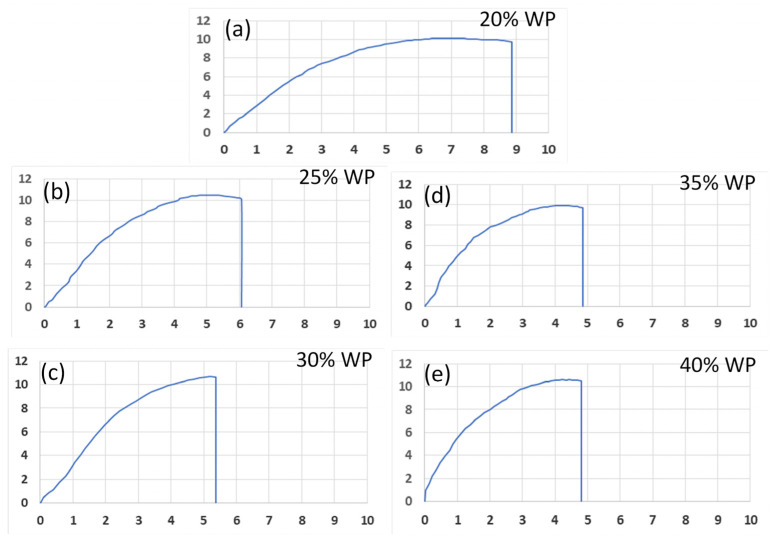
Stress–strain diagram of samples with different WP percentages: (**a**) 20 wt.% WP; (**b**) 25 wt.% WP; (**c**) 30 wt.% WP; (**d**) 35 wt.% WP; and (**e**) 40 wt.% WP.

Figure 5 shows the stress–strain diagram of samples with different WP percentages. The area below the stress–strain curves reduces gradually when the WP percentage increases from 20 wt.% to 40 wt.%. Notably, from 20 wt.% to 30 wt.% WP, this area reduces rapidly. From 30 wt.% to 40 wt.%, the reduction speed is slower. These results are consistent with the UTS and elongation results, which are presented in Figure 2 and Figure 3. The reduction of the area below the stress–strain when increasing the WP content also indicates the reduction in the toughness of the WPC when increasing the WP content.

In general, the UTS value of the WPC changes slightly around 10.4 MPa when changing the WP content from 20 wt.% to 40 wt.%. The elongation at break value reaches the highest value of 7.40 wt.% at 20 wt.% WP, while the elastic modulus obtains the highest value of 433.6 MPa at 30 wt.% WP. Notably, the WPC with 25 wt.% WP has good tensile characteristics with a UTS value of 10.42 MPa, an elongation value of 5.53 MPa, and an elastic modulus value of 364.7 MPa.

### 3.2. Flexural Test Results

**Figure 6:**
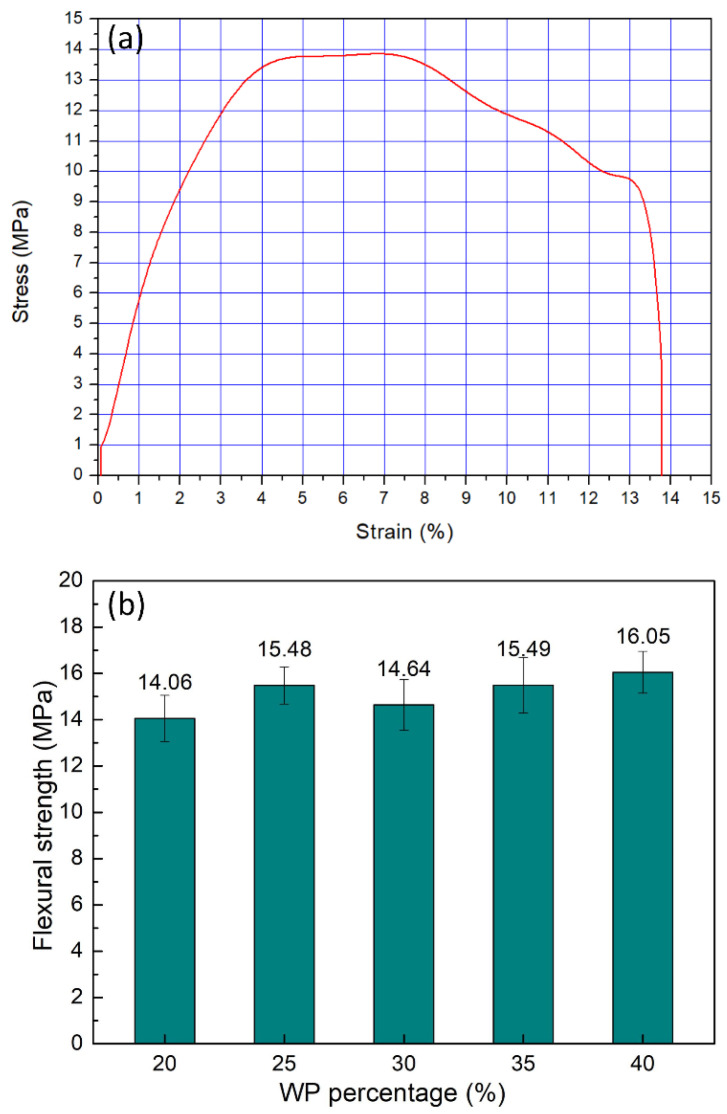
Stress–strain diagram and flexural strength comparison of WPC at different WP percentages: (**a**) stress–strain diagram of sample with 20 wt.% WP; (**b**) UTS comparison diagram.

The stress–strain diagram and flexural strength comparison of WPC at different WP percentages are displayed in Figure 6. The flexural strength values are 14.06 MPa, 15.48 MPa, 14.64 MPa, 15.49 MPa, and 16.0 MPa. Generally, increasing the WP leads to an improvement in the flexural strength of the WC. The WPC has the highest flexural strength of 16.0 MPa at 40 wt.% WP, while at 20 wt.% WP, the WPC has the lowest flexural strength of 14.06 MPa. Overall, the flexural strength value oscillates gently around 15 MPa. This result is similar to the UTS value, which also indicates that the WP content has a slight impact on the UTS and flexural strength. However, the flexural strength value has a higher deviation value, indicating that WP content has a stronger impact on flexural strength than the UTS. Kaymakci et al. [40] mixed PP with 50 wt.% rice husk powder, achieving a flexural strength of 11.27 MPa, which is lower than this study’s results. Valles-Rosales et al. [41] achieved a flexural strength of 16.2 MPa when mixing PP with 42.5 wt.% chili-stem waste particles.

**Figure 7:**
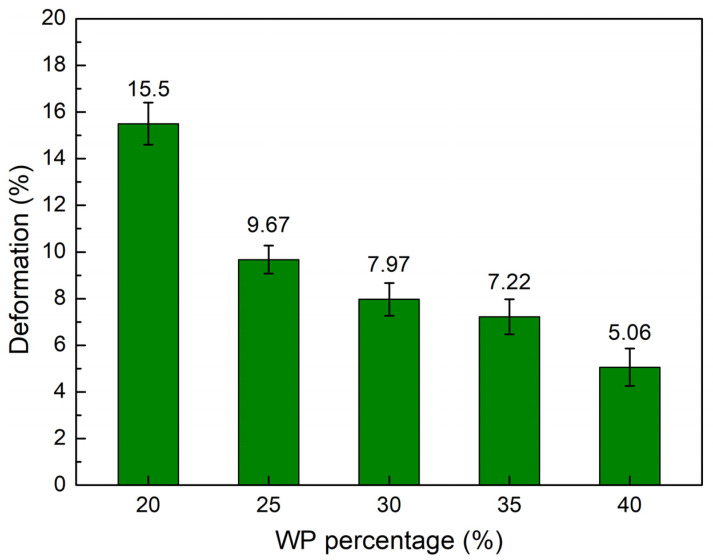
Maximum flexural strain at break values comparison of WPC at different WP percentages.

The maximum flexural strain at break comparison diagram of the WPC at different WP percentages is presented in Figure 7. The elongation values of the WPC samples are 15.50 wt.%, 9.67 wt.%, 7.97 wt.%, 7.22 wt.%, and 5.06 wt.%, corresponding to 20 wt.%, 25 wt.%, 30 wt.%, 35 wt.%, and 40 wt.% WP. Interestingly, adding more WP to the WPC leads to a gradual decrease in the maximum flexural strain value. At 20 wt.% WP, the WPC has the highest maximum flexural strain value of 15.50 wt.%. It declines to the lowest value of 5.06 wt.% when the WP content increases to 40 wt.%. The reason is that the WP does not have as much ductility as the PP matrix; therefore, adding more WP results in a reduction in the maximum flexural strain at break value.

**Figure 8:**
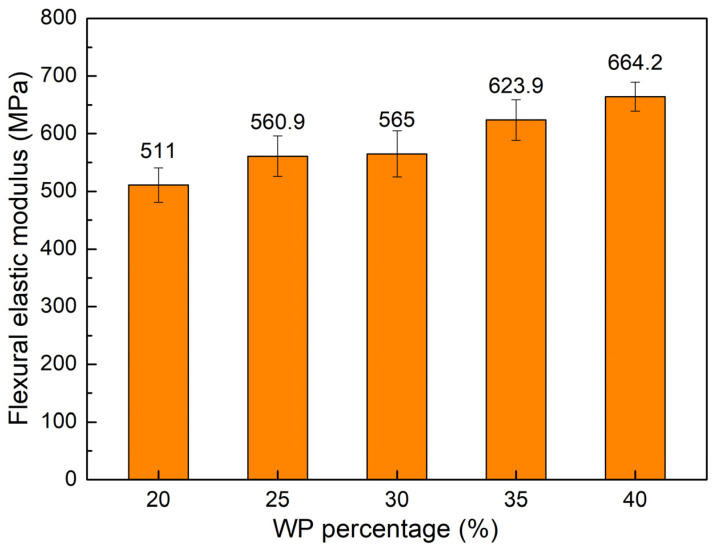
Flexural elastic modulus values comparison of WPC at different WP percentages.

Figure 8 exhibits the flexural elastic modulus value comparison of WPC at different WP percentages. The elastic modulus values of the WPC are 511 MPa, 560.9 MPa, 565 MPa, 623.9 MPa, and 664.2 MPa, corresponding to 20 wt.%, 25 wt.%, 30 wt.%, 35 wt.%, and 40 wt.% WP. Increasing the WP content gives rise to the flexural elastic modulus value, which is reversed to the maximum flexural strain value. This result is consistent with Bessa et al.’s study [42], which also indicated that increasing the WP content leads to an improvement in the flexural elastic modulus of the WPC. However, increasing the WP content mostly leads to a reduction in the elongation at break and elastic modulus, as mentioned in the tensile test.

**Figure 9:**
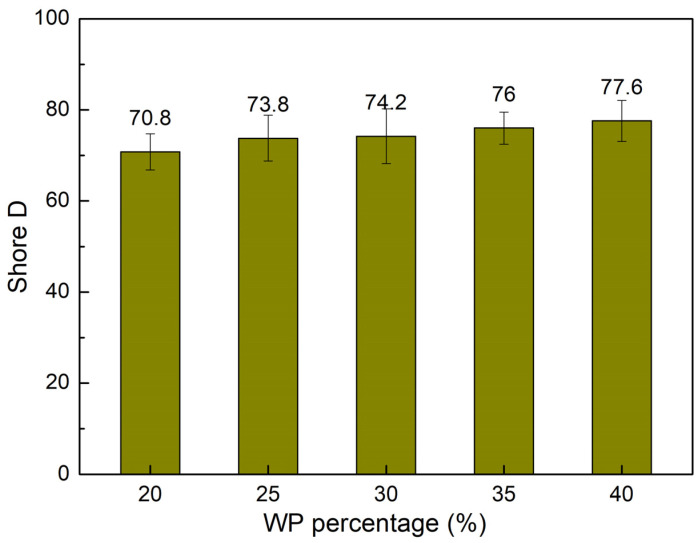
Shore D hardness value comparison of WPC at different WP percentages.

Figure 9 shows the shore D hardness value comparison of WPC at different WP percentages. The shore D hardness values of the WPC are 70.8, 0.76 wt.%, 0.97 wt.%, 1.17 wt.%, and 1.58 wt.%, corresponding to 20 wt.%, 25 wt.%, 30 wt.%, 35 wt.%, and 40 wt.% WP. Increasing the WP content gives rise to the hardness of the WPC due to the higher hardness of the WP compared to the PP matrix. This is also the reason why increasing the WP content leads to a reduction in the maximum flexural strain and an increase in the flexural elastic modulus, as shown in Figure 7 and Figure 8. This result is consistent with the Teymoorzadeh et al. [43] study, which also investigated the impact of WPC on hardness. In that study, increasing the WP from 0 wt.% to 40 wt.% led to a gradual increase in shore D hardness from 79 to 89 of the polylactic acid wood composite.

Overall, the flexural strength of the WPC varies around 15 MPa when the WP increases from 20 wt.% to 40 wt.%. The increase in the WP mainly leads to increased flexural strength. The flexural elastic modulus also increases when the WP content increases. On the contrary, the maximum flexural strain decreases when the WP content increases. The maximum flexural strain value achieves the highest number of 15.50 wt.% at 20 wt.% WP, while the flexural elastic modulus reaches the highest value of 664.2 MPa at 40 wt.% WP. Remarkably, if the maximum flexural strain is not critical, WPC samples with 40 wt.% WP are a good selection due to their good flexural strength of 16.0 MPa and flexural elastic modulus of 664.2 MPa. The highest shore D hardness of 77.6 of the WPC is obtained at 40 wt.% WP. Increasing the WP content will increase the hardness of the WPC.

### 3.3. SEM, DSC and Water Absorption Tests Results

**Figure 10:**
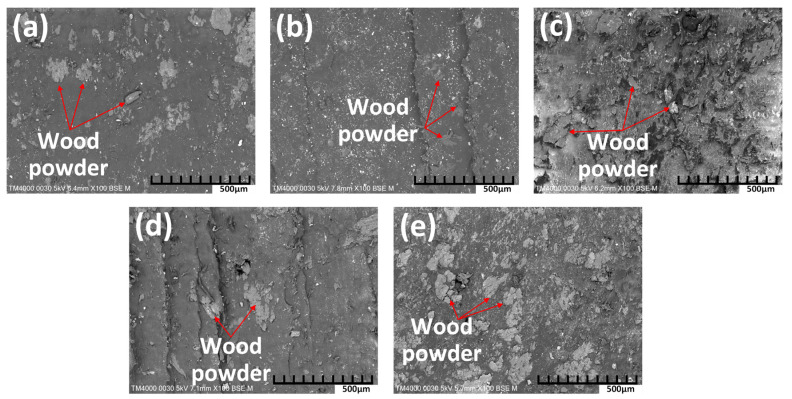
SEM figures of WPC at different WP percentages: (**a**) 20 wt.% WP; (**b**) 25 wt.% WP; (**c**) 30 wt.% WP; (**d**) 35 wt.% WP; and (**e**) 40 wt.% WP.

Figure 10 shows the SEM figures of WPC at different WP percentages. Before SEM testing, the samples are not coated with gold or platinum. With the compatibilizer Olenamide 5 wt.%, all WPC samples present good bonding between the WP and PP matrix. There are no separations between WP and PP matrix, indicating the high quality of the samples. The WP size varies around 90.2 µm–112.9 µm, which is a fine WP. Moreover, the WP was distributed evenly on the composite matrix, indicating the good effect of the compatibilizer Olenamide. Remarkably, with the presence of the compatibilizer Olenamide, increasing the WP content from 20 wt.% to 40 wt.% does not lead to the accumulation of the WP in the composite matrix. Ratanawilai et al. [44] indicated that reducing the average WP size from 315 µm to 180 µm led to a slight increase in the tensile and flexural strength. In this study, the WP size is smaller than that report, indicating that the WP size is small enough to generate a good WPC.

**Figure 11:**
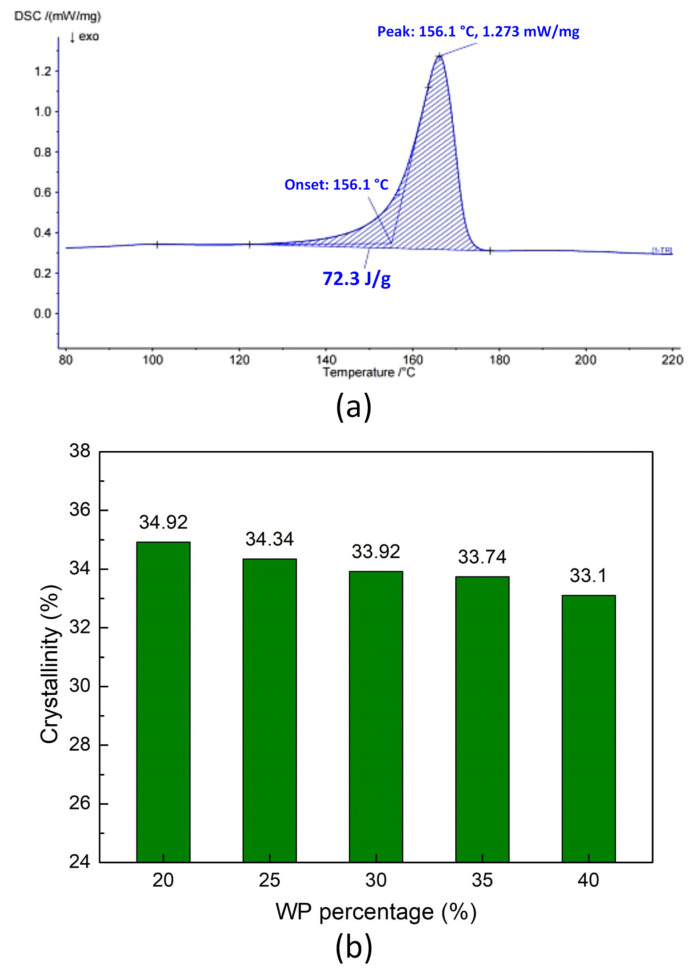
DSC results of WPC at different WP percentages: (**a**) DSC curve of sample with 20 wt.% WP; (**b**) crystallinity comparision.

DSC results of WPC at different WP percentages are presented in Figure 11. Each sample composition uses about 15 mg to conduct the test. The crystallinity is calculated by the following equation:

(1) $$X=\frac{{\Delta H}_{m}}{{\Delta H}_{m0}}\times 100\;wt.\%,$$

where ΔH_m_ is melting enthalpy; the ΔH_m0_ of PP is 207 J/g [45].

The crystallinity values of the WPC are 34.92 wt.%, 34.34 wt.%, 33.92 wt.%, 33.74, and 33.1 wt.%, corresponding to 20 wt.%, 25 wt.%, 30 wt.%, 35 wt.%, and 40 wt.% WP. The crystallinity of the WPC reduces gradually and slightly when the WP content increases. The presence of coconut WP, which has an amorphous structure, leads to a lower crystallinity percentage of the WPC. However, if the true fraction of PP is considered during the crystallinity calculation, meaning the WP is not counted, the crystallinity values are higher. The crystallinity values of the PP matrix are 43.65 wt.%, 45.79 wt.%, 48.46 wt.%, 51.91, and 55.17 wt.%, corresponding to 20 wt.%, 25 wt.%, 30 wt.%, 35 wt.%, and 40 wt.% WP. It means that the PP matrix has a higher crystallinity when increasing the WP in the WPC.

**Figure 12:**
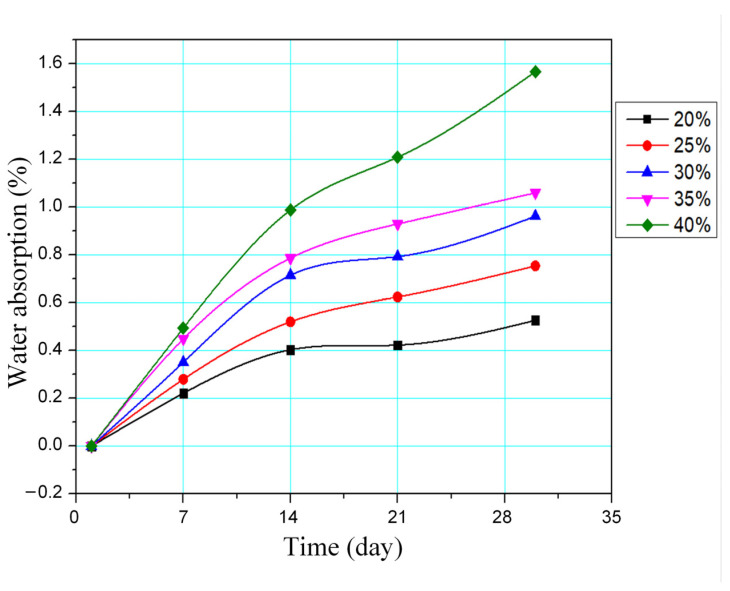
Water absorption of WPC at different WP percentages.

Figure 12 shows the water absorption of WPC at different WP percentages. Water absorption measurements are tested via the standard ASTM D 570-88. Water absorption is calculated following equation:

(2) $$X=\frac{W_{1}-W_{0}}{W_{0}}\times 100\;wt.\%$$

where W_1_ is the weight of the sample after water absorption and W_0_ is the original weight of the samples.

The maximum water absorption of WPC after 30 days is 0.52 wt.%, 0.76 wt.%, 0.97 wt.%, 1.17 wt.%, and 1.58 wt.%, corresponding to 20 wt.%, 25 wt.%, 30 wt.%, 35 wt.%, and 40 wt.% WP. Increasing the WP content leads to an improvement in the water absorption of the WPC, which is similar to Bessa et al.’s report [25]. The reason is the WP has a strong water absorption; therefore, increasing its content will result in a better water absorption of the WPC. Moreover, the water absorption process for 30 days could be divided into two stages. In the first stage, from 0 to the 14th day, the water absorption rises rapidly. Then, in the second stage, from the 14th day to the 30th day, the water absorption speed reduces as the WP becomes saturated. In general, the WPC samples have a low water absorption capacity.

### 3.4. Optimization by Taguchi Method

In this section, the study aims to optimize the tensile strength and flexural strength of the WPC using the Taguchi method. In Table 2, the injection parameters and WP are calculated following the Taguchi method via Minitab software version 20.3 with L25 orthogonal array, four factors, and five levels.

Table 3 presents the response table for means of coconut WPC for the UTS value. The results show that WP content plays the most important role in the UTS value of the coconut WPC. The filling pressure has the second rank, followed by the packing pressure. Finally, the surveyed melt temperature does not impact the UTS value as strongly as the other parameters. Therefore, controlling the WP content could lead to a suitable selection when using the WPC.

**Figure 13:**
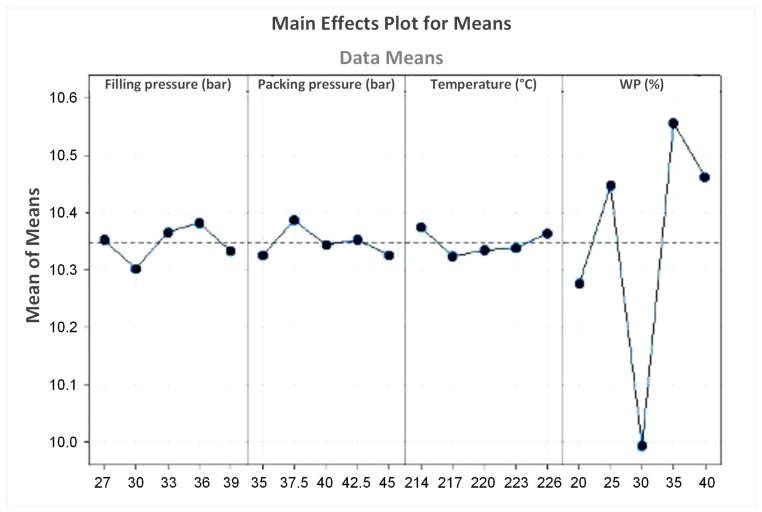
Main effects plot for means of coconut WPC for the UTS value.

Figure 13 presents the main effects plot for means of coconut WPC for the UTS value. The optimal parameters that could lead to the highest UTS value are a filling pressure of 36 bar, a packing pressure of 37.5 bar, a melt temperature of 214 °C, and a WP content of 35 wt.%. The predicted UTS value that applied these parameters is 10.658 MPa.

Table 4 represents the response table for means of coconut WPC for the flexural strength value. Similar to the UTS value, WP content also has the highest impact on the flexural strength value of the coconut WPC. The melt temperature has the second position on the impact range of the flexural strength value, followed by the packing pressure. Notably, the filling pressure has the weakest impact on the flexural strength value. This is different from the UTS value, in which the filling pressure ranks second place. Overall, the WP content is the most important parameter when considering both the UTS and the flexural strength values.

**Figure 14:**
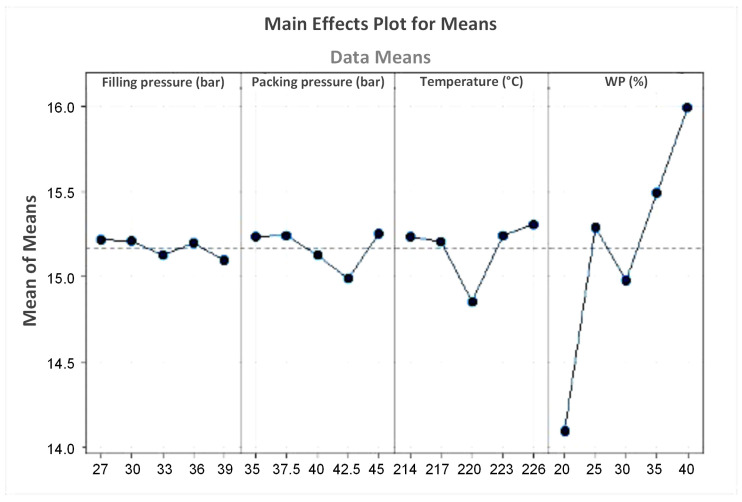
Main effects plot for means of coconut WPC for the flexural strength value.

Figure 14 shows the main effects plot for means of coconut WPC for the flexural strength value. The optimal parameters are a filling pressure of 27 bar, a packing pressure of 45 bar, a melt temperature of 226 °C, and a WP content of 40 wt.%. The predicted flexural strength value that applied these parameters is 10.464 MPa.

## 4. Conclusions

This study studies the mechanical properties of coconut sawdust powder mixed with PP. The impact of compatibility content, WP content, and injection molding parameters on the characteristics of the coconut WPC is surveyed. Some important results are as follows:

- Depending on the coconut WP content and the desired properties, the mechanical properties of the WPC would achieve their best results at different WP contents. The highest UTS value is achieved at 35 wt.% WP. The elongation reaches the highest value of 7.40 wt.% at 20 wt.% WP, while the elastic modulus obtains the highest value of 433.6 MPa at 30 wt.% WP.
- The flexural strength, the flexural elastic modulus, and the shore D hardness have a linear relation to the WP content. An increase in WP content will increase these properties, reaching their best properties at 40% WP.
- SEM results show that all WPC samples present good bonding between the WP and the PP matrix. Moreover, the WP is distributed evenly on the composite matrix due to the presence of the compatibilizer.
- The crystallinity of the WPC reduces gradually and slightly when the WP content increases, indicating that the WP does not strongly impact the WPC crystallinity.
- Taguchi’s results show that WP content plays the most important role in the UTS value of the coconut WPC. The filling pressure has the second rank, followed by the packing pressure. Finally, the surveyed melt temperature does not strongly impact the UTS value like the other parameters. Therefore, controlling the WP content could lead to a suitable selection when using the WPC. Further studies should focus on WPCs with a coconut WP percentage greater than 45%.

## Figures and Tables

**Table 1:** Characteristics of Advanced-PP 1100 N.

Properties	Unit	Test Method	Value
Melt flow rate (230 °C/2.16 KG)	g/10 min	ISO 1133 [30]	12
Tensile modulus of elasticity	MPa	ISO 527-2 [31]	1550
Tensile stress at yield	MPa	ISO 527-2	35
Tensile strain at yield	%	ISO 527-2	8
Tensile strain at break	%	ISO 527-2	>50
Charpy impact strength notched	kJ/m^2^	ISO 179/1eA [32]	3.0
Ball indentation hardness (H 358/30)	MPa	ISO 2039-1 [33]	78
Melting point, DSC	°C	ISO 3146 [34]	163
Heat deflection temperature—HDT/B (0.45 Mpa)	°C	ISO 75-2 [35]	85
Vicat softening temperature—VST/A50 (10 N)	°C	ISO 306 [36]	154
Density	g/cm^3^	ISO 1183 [37]	0.91

**Table 2:** Injection parameters and WP content of coconut WPC.

Sample No.	Filling Pressure (Bar)	Packing Pressure (Bar)	Melt Temperature (°C)	Powder Content (wt.%)
1	27	35	214	20
2	27	37.5	217	25
3	27	40	220	30
4	27	42.5	223	35
5	27	45	226	40
6	30	35	217	30
7	30	37.5	220	35
8	30	40	223	40
9	30	42.5	226	20
10	30	45	214	25
11	33	35	220	40
12	33	37.5	223	20
13	33	40	226	25
14	33	42.5	214	30
15	33	45	217	35
16	36	35	223	25
17	36	37.5	226	30
18	36	40	214	35
19	36	42.5	217	40
20	36	45	220	20
21	39	35	226	35
22	39	37.5	214	40
23	39	40	217	20
24	39	42.5	220	25
25	39	45	223	30

**Table 3:** Response table for means of coconut WPC for the UTS value.

Level	Filling Pressure (Bar)	Packing Pressure (Bar)	Melt Temperature (°C)	WP Content (wt.%)
1	10.352	10.326	10.374	10.276
2	10.302	10.386	10.324	10.446
3	10.366	10.344	10.334	9.994
4	10.382	10.352	10.338	10.556
5	10.332	10.326	10.364	10.462
Delta	0.080	0.060	0.050	0.562
Rank	2	3	4	1

**Table 4:** Response table for means of coconut WPC for the flexural strength value.

Level	Filling Pressure (Bar)	Packing Pressure (Bar)	Melt Temperature (°C)	WP Content (wt.%)
1	15.22	15.23	15.24	14.10
2	15.21	15.24	15.21	15.29
3	15.13	15.13	14.86	14.98
4	15.20	14.99	15.24	15.49
5	15.10	15.26	15.31	15.99
Delta	0.12	0.27	0.45	1.89
Rank	4	3	2	1

## Data Availability

The data used to support the findings of this study are available from the corresponding author upon request.
